# Propensity for risky choices despite lower cue reactivity in adolescent rats

**DOI:** 10.3389/fnbeh.2023.1297293

**Published:** 2023-11-20

**Authors:** Sandford Zeng, Elin F. B. McLaughlin, Aishwarya Ramesh, Sara E. Morrison

**Affiliations:** Department of Neuroscience, University of Pittsburgh, Pittsburgh, PA, United States

**Keywords:** adolescent, gambling, risk, sign tracking, goal tracking, reward, cue, Pavlovian conditioning

## Abstract

Adolescence is a time of heightened risk-taking across species. Salient audiovisual cues associated with rewards are a common feature of gambling environments and have been connected to increased risky decision-making. We have previously shown that, in adult male rats, sign tracking – a behavioral measure of cue reactivity – predicts an individual’s propensity for suboptimal risky choices in a rodent gambling task (rGT) with win-paired cues. However, adolescents perform less sign tracking than adult animals, suggesting that they are less cue-reactive than adults in some circumstances. Therefore, we investigated the performance of adolescent male rats on the rGT with win cues and examined its relationship with their sign-tracking behavior. We found that adolescents make more risky choices and fewer optimal choices on the rGT compared with adults, evidence of the validity of the rGT as a model of adolescent gambling behavior. We also confirmed that adolescents perform less sign tracking than adults, and we found that, unlike in adults, adolescents’ sign tracking was unrelated to their risk-taking in the rGT. This implies that adolescent risk-taking is less likely than that of adults to be driven by reward-related cues. Finally, we found that adults trained on the rGT as adolescents retained an adolescent-like propensity toward risky choices, suggesting that early exposure to a gambling environment may have a long-lasting impact on risk-taking behavior.

## Introduction

Excessive gambling, like many maladaptive behaviors, can be exacerbated by environmental cues linked to rewards. Gambling environments are typically rich in salient audiovisual cues, and it has been shown that such cues play a role in the development of pathological gambling (Grant and Bowling, 2015; Barrus et al., 2016). Individuals can vary widely in their sensitivity to reward-related cues, and cue reactivity has been established as a factor in an individual’s vulnerability to gambling disorder (GD; Barrus et al., 2016; Nautiyal et al., 2017) and other neuropsychiatric conditions, including substance use disorders (Hellberg et al., 2019; Hill-Bowen et al., 2021).

Sign tracking (Hearst and Jenkins, 1974) is a readily measurable behavior that can quantify cue reactivity among both humans (Colaizzi et al., 2020; Schad et al., 2020) and non-human animals, making it particularly useful for translational and comparative studies. In a typical rodent sign-tracking paradigm, a cue (e.g., an extended lever) predicts the delivery of a reward (e.g., a sugar pellet) in a separate location. After training on this paradigm, some subjects will approach and interact with the cue – a sign-tracking response – while others will preferentially approach the site of reward delivery, a behavior known as goal tracking (Boakes, 1977). A propensity for sign tracking over goal tracking has been linked to various forms of impulsivity (Flagel et al., 2010; Lovic et al., 2011; Yager and Robinson, 2013) as well as substance abuse-related behaviors such as drug-seeking and relapse (Saunders and Robinson, 2013; Versaggi et al., 2016). Furthermore, we have recently shown that sign tracking predicts suboptimal behavior on a rodent gambling task (rGT) featuring salient audiovisual “win cues” (adapted from Barrus and Winstanley, 2016), including increased choice of a high-risk, high-reward option. Similarly, another measure of cue reactivity, conditioned orienting, predicts both impulsivity and risky decision-making in a task that pairs larger rewards with aversive consequences (foot shock; Olshavsky et al., 2014).

Adolescence is known to be a time of heightened risk-taking in both humans and non-human animals (Steinberg, 2008; Doremus-Fitzwater et al., 2010; Van Leijenhorst et al., 2010; Zoratto et al., 2013; Westbrook et al., 2018); it is also characterized by certain forms of impulsivity (Burton and Fletcher, 2012) and poor decision-making (Steinberg et al., 2008). Excessive gambling, including diagnosis of GD, is on the rise among adolescents (Calado et al., 2017; Dowling et al., 2017; Richard and King, 2023), abetted by the emergence of novel forms of gambling such as online sports betting (Stefanovics et al., 2023) and video games with gambling elements (Hing et al., 2023). Prevalence estimates for problem gambling among adolescents vary widely among studies with different methodologies, with an average around 2.3% (Dowling et al., 2017). Surprisingly, however, few studies have examined the behavior of adolescent animals in a gambling setting comparable to the rGT. The rGT represents a rodent version of the Iowa Gambling Task (IGT), which provides a measure of maladaptive risk-taking in humans, and it has been well validated in adult rats (Winstanley and Clark, 2016). Because human younger adolescents perform poorly on the IGT (Marquez-Ramos et al., 2023), we hypothesized that adolescent rats would also perform less optimally than adults. If so, the rGT could provide a translational model of adolescent gambling and perhaps risky decision-making more generally.

At the same time, adolescents exhibit marked differences from adults in some aspects of cue reactivity. By some measures, adolescents show enhanced behavioral and neural responses to reward-paired cues (Burton et al., 2011; Sturman and Moghaddam, 2011; Marshall et al., 2020), although this may vary depending on whether a task involves Pavlovian or instrumental conditioning (McCane et al., 2021), among other factors. On the other hand, we and others have shown that adolescent rats are less prone to sign tracking and more prone to goal tracking in response to a Pavlovian cue compared with adults (Doremus-Fitzwater and Spear, 2011; Anderson et al., 2013; Rode et al., 2019). This may be related to the enhanced behavioral flexibility and exploratory drive of adolescents, relative to adults (Simon et al., 2013; Westbrook et al., 2018) – attributes that may also contribute to adolescents’ relatively weak habit formation (Serlin and Torregrossa, 2015) despite their greater reward sensitivity (Doremus-Fitzwater and Spear, 2016; Marshall et al., 2017).

In adults, risky decision-making on a task such as the rGT with win cues (Barrus et al., 2015; Barrus and Winstanley, 2016) seems to be at least partially driven by the influence of reward-paired cues, as mediated by an individual’s trait cue reactivity (Swintosky et al., 2021). However, adolescents are less likely than adults to transfer incentive salience from a reward to a cue, as measured by their sign-tracking behavior, while at the same time, they show enhanced risk-taking across species by a number of measures. Thus, it remains unclear whether adolescents will show a similar relationship to adults between sign tracking and risky decision-making on the rGT. In order to address this question, we trained adolescent subjects on a Pavlovian conditioned approach (PCA) task followed by the rGT with win cues and compared their behavior on both tasks with a preexisting data set from adult rats (Swintosky et al., 2021). We also examined the influence of early exposure to a gambling task on subsequent adult performance on the rGT with win cues.

## Materials and methods

All procedures were performed in accordance with the standards of the National Institutes of Health and were approved by the Institutional Animal Care and Use Committee of the University of Pittsburgh.

### Subjects

Adolescent subjects were 64 male Long-Evans rats obtained from Charles River at age 21 days. The adult comparison group (see Swintosky et al., 2021) comprised 64 male Long-Evans rats obtained from Charles River at an initial weight of 275–300 g (approximately 9 weeks of age). Subjects were allowed to acclimate to the housing facility for 5–7 days, then gently habituated to handling over at least two sessions prior to the initiation of food restriction and training. All subjects were housed in pairs throughout the study. Rats were placed on mild food restriction (10 g per day for adolescents, 14 g for adults) two days before the start of training and remained on food restriction throughout training. Adolescents who were retested as adults were provided food *ad libitum* for ~4 weeks in between tests and restarted on food restriction at least 2 days before retesting. Subjects were weighed regularly to ensure they did not fall below ~85% of free-feeding weight (adults) or the weight of age-matched free-feeding controls (adolescents).

### Apparatus and training

Behavioral training and testing took place in 8 standard operant chambers (Coulbourn Instruments) equipped with a house light, food magazine, and three illuminable nosepoke operanda recessed into the wall opposite the food magazine. A retractable lever was located to the side of the food magazine (left or right counterbalanced) and a white cue light was located above the lever. A speaker for delivering auditory cues was located above the food magazine. Rewards were 45 mg sucrose pellets (Bio-Serv). All subjects were initially trained to retrieve pellets from the food magazine over 2 daily sessions in which 50 pellets were delivered over a variable time schedule averaging 60 s.

### Pavlovian conditioned approach

Following magazine training, subjects were trained for 7 sessions on a Pavlovian conditioned approach task that typically elicits sign tracking and/or goal tracking behavior (Rode et al., 2019; Swintosky et al., 2021). Sessions consisted of 25 cue presentations with intertrial intervals (ITIs) drawn from a truncated exponential distribution averaging 30 s. Cues consisted of an 8 s extension of the lever and illumination of the flashing (5 Hz) cue light, immediately followed by delivery of the sucrose pellet to the food magazine. No action was required for reward delivery. Nosepokes were covered during Pavlovian conditioned approach training.

### Rodent gambling task with win cues

The rodent gambling task (rGT) with win cues was based on a similar task used by Barrus and Winstanley (2016) which we have previously adapted (Swintosky et al., 2021). Due to time constraints, adolescent subjects began training for the rGT the day after completion of Pavlovian conditioned approach training. In all other respects, training of adolescents was identical to that of adults. First, subjects were given 5 daily sessions of nosepoke training, in which one nosepoke would be illuminated after an ITI of 15 s. If the rat entered the nosepoke within 10 s, one sucrose pellet would be delivered to the food magazine; if not, the nosepoke light would extinguish and the ITI would restart. Nosepokes were illuminated pseudo-randomly such that the rat entered all nosepokes equally during the session. Sessions were terminated when 100 rewards were obtained or after 1 h. All subjects completed the task within 1 h by the end of training.

Next, rats were given 7 sessions of training on a “forced choice” version of the rGT. In these sessions, the nosepoke contingencies were identical to the full task, but only one nosepoke was available (illuminated) on each trial. Entries into the non-illuminated nosepokes resulted in a brief (2 s) timeout (indicated by extinguishing the house light) followed by a restart of the 10 s ITI. Entries into the illuminated nosepoke resulted in delivery of the specific reward (with win cues) or timeout associated with that nosepoke in the rGT. In this way, rats were exposed to all the nosepoke contingencies equally. Sessions were 40 min.

Finally, adolescent rats were given 7 sessions on the full version of the rGT (adults in the comparison group (Swintosky et al., 2021) were given at least 7 sessions and no more than 9). Each session lasted 40 min and the ITI was 10 s. On each trial, all three nosepokes were illuminated; if the rat entered a nosepoke within 10 s, the reward (with win cues) or timeout associated with that nosepoke would be delivered. If no nosepoke was selected, the ITI was restarted and the trial was scored as an omission. Premature entries (during the ITI) resulted in a brief (2 s) timeout indicated by extinguishing the house light followed by a restart of the ITI.

**Figure 1:**
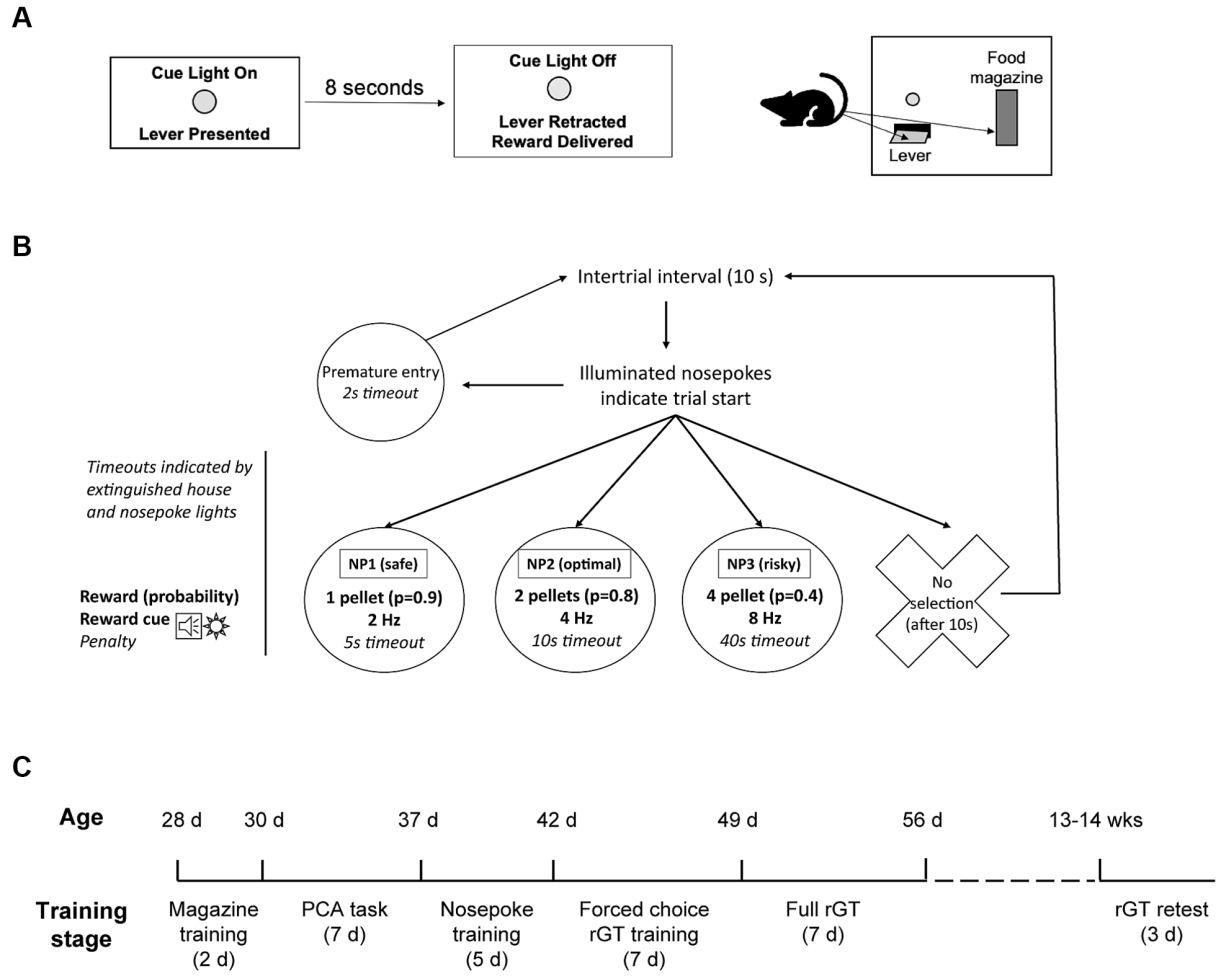
Task structures and timeline. **(A,B)** Task structure of the Pavlovian conditioned approach (PCA) task and the rodent gambling task (rGT) with win cues. In the PCA task **(A)**, behavior directed toward the lever constitutes sign tracking while behavior directed toward the food magazine constitutes goal tracking. In the rGT **(B)**, trials are initiated by the illumination of all three nosepoke lights. Each nosepoke light is associated with a different probability and size of reward (sugar pellets) and aversive outcome (timeout). Rewards are accompanied by win cues of varying salience (frequency and/or variety). **(C)** Training timeline for adolescent subjects (*n* = 64). Ages are modes and could vary 1–2 days from age listed. A subset of subjects (*n* = 32) were retested as adults age 13–14 weeks, similar to adult subjects trained on the same tasks (Swintosky et al., 2021).

A schematic of the rGT task design is shown in Figure 1. The nosepoke contingencies and locations were the same for all animals. Nosepoke 1 (NP1), located away from the chamber door, was the “safe” choice: it was associated with a 90% probability of delivery of 1 pellet, along with a “boring” win cue of slow flashing of the NP1 light (2 Hz) and an intermittent tone (2 Hz, tone frequency 500 Hz). There was also a 10% chance of a short (5 s) timeout. Nosepoke 2 (NP2), located in the middle, was the optimal choice. It was associated with an 80% probability of delivery of 2 pellets, along with a moderate win cue, consisting of flashing of the NP2 light (4 Hz) and an intermittent tone played concurrently (4 Hz). There was also a 20% chance of a medium (10 s) timeout. Nosepoke 3, located nearest the chamber door, was the risky choice. It was associated with a 40% probability of delivery of 4 pellets, along with an “exciting” win cue (high salience, high variability). The exciting win cue consisted of one of 4 possible sequences of flashing nosepoke lights (8 Hz) along with a fast intermittent tone (8 Hz). There was also a 60% probability of a long timeout (40 s). All timeouts were indicated by extinguishing the house light.

Adolescent rats were trained and tested sequentially in four cohorts of 16. The last two cohorts of adolescent-trained animals (*n* = 32) were retested on the rGT after reaching adulthood, at an interval of 4–5 weeks after their final rGT session as adolescents. Thus, these rats were age 13–14 weeks when retested, approximately the same age as the adult-trained rats at the completion of their training. Retesting consisted of 3 consecutive daily sessions on the standard rGT with win cues. Behavior was averaged over these 3 sessions for analysis.

### Analysis of behavior

All analyses were carried out using custom-written functions in Matlab (Mathworks). Sign tracking and goal tracking behavior were quantified using the PCA index (Meyer et al., 2012), which consists of the average of three ratios: (1) the probability index, which compares the probability of lever pressing vs. magazine entry during the cue, calculated as (P_lev_ – P_mag_), (2) the bias index, which compares the number of lever presses vs. magazine entries per cue, calculated as (#lever - #magazine / #lever + #magazine), and (3) the latency index, which compares the latency from cue onset to lever press vs. magazine entry, calculated as (latency_lev_ – latency_mag_ / cue length). For trials in which a behavior was not performed, the latency is defined as the cue length (8 s). All of these indices, including the PCA index, range from −1.0 to +1.0, with negative numbers indicating a preference for goal tracking and higher numbers a preference for sign tracking. We operationally define sign trackers as individuals with a PCA index >0.25 and goal trackers as individuals with a PCA index < −0.25. PCA index is averaged over the last three days of training on the Pavlovian conditioned approach task.

For the rGT, behavioral measures analyzed include percent choice of each option (NP1, 2, or 3), number of rewards obtained, number of premature nosepoke entries, and number of omitted trials. Percentages were arcsine transformed to avoid ceiling effects. Choice data was analyzed using repeated measures one-way ANOVA with choice percentage as a within-subjects factor and age status (adolescent or adult) as a between-subjects factor. Post-hoc comparisons were corrected using the Dunn-Sidak method; significance level was set at α = 0.05. Correlations are reported using Spearman’s rho (r_s_).

## Results

We trained 64 adolescent male rats sequentially on two behavioral tasks: first, a Pavlovian conditioned approach task (Figure 1A), and second, a rodent gambling task (rGT) with win cues (Figure 1B). The Pavlovian conditioned approach task was a paradigm that we have previously used to elicit sign-tracking and goal-tracking behavior in both adolescents and adults (Rode et al., 2019; Swintosky et al., 2021). In this task, rats are exposed to cues comprised of lever extension and flashing cue light, followed by a reward (sugar pellet) delivered to a food magazine. Sign-tracking behavior is represented by lever deflections, and goal-tracking behavior by magazine entries during the 8 s cue. We quantified sign-tracking and goal-tracking behavior using a composite measure, the PCA (Pavlovian conditioned approach) index (Meyer et al., 2012), which ranges from −1.0 (all goal tracking, no sign tracking) to +1.0 (all sign tracking, no goal tracking).

**Figure 2:**
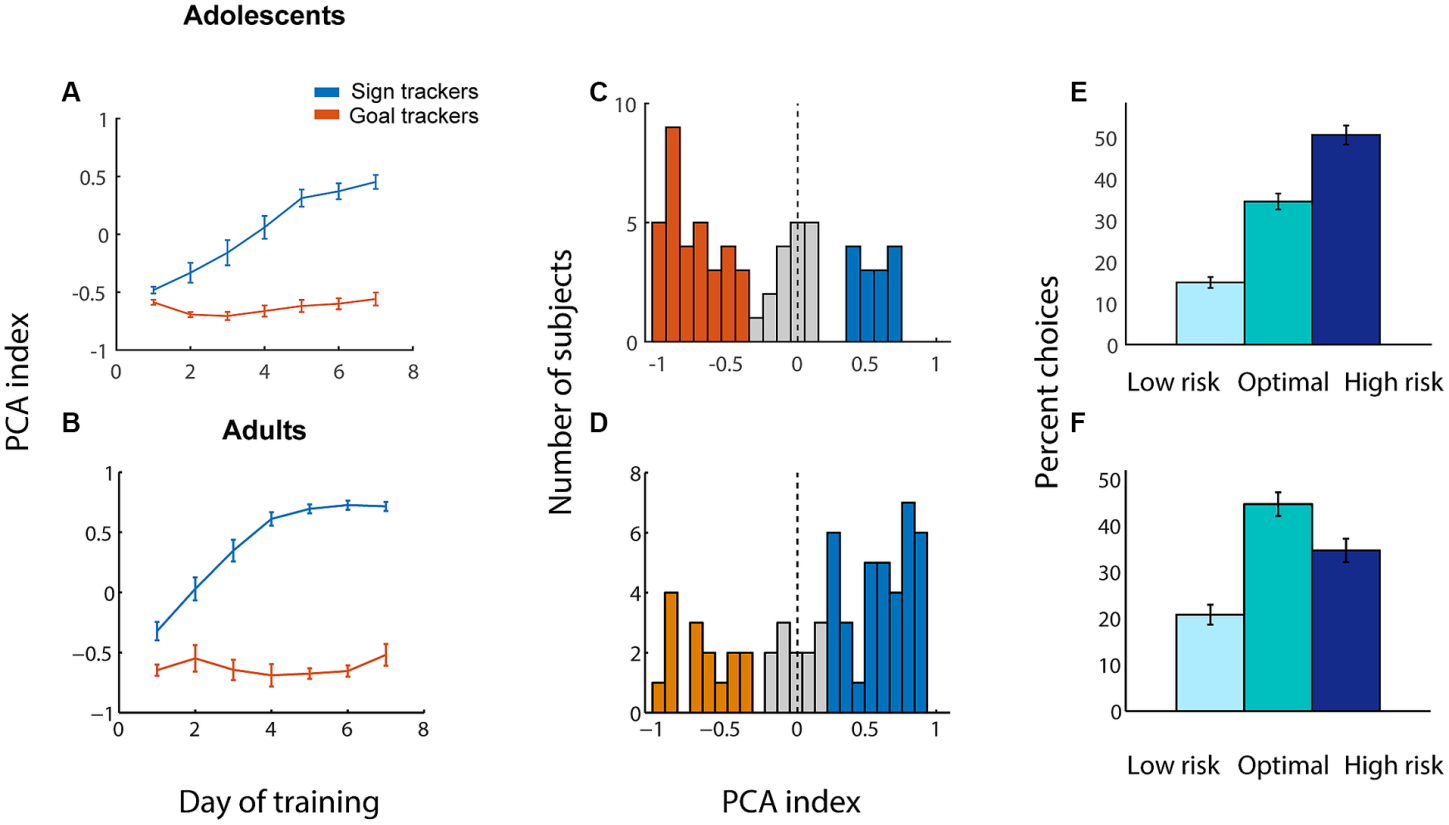
Adolescents perform less sign tracking than adults but make more risky choices on the rGT with win cues. **(A,B)** Average Pavlovian conditioned approach (PCA) index among sign trackers (blue) and goal trackers (orange) for each of the 7 days of training for adolescents **(A)** and adults **(B)**. Higher PCA index indicates more sign tracking relative to goal tracking. **(C,D)** Average PCA index for adolescents **(C)** and adults **(D)** over the last 3 days of training. Orange, individuals categorized as goal trackers; blue, sign trackers; gray, intermediate. (E, F) Average choice distribution among adolescents **(E)** and adults **(F)** over the last 3 days of training on the rGT with win cues. All panels, error bars indicate SEM.

Adolescent subjects underwent 7 days of training on the Pavlovian conditioned approach task (Figure 2A). Similar to their adult counterparts (Figure 2B), a subset of adolescent subjects developed a tendency toward sign tracking over the course of training, while others retained a preference for goal tracking (adult data used here was originally reported in Swintosky et al., 2021). Distributions of PCA index at the end of training (averaged over the last three sessions) are shown in Figure 2C (for adolescents) and Figure 2D (for adults). As we and others have previously shown (Rode et al., 2019), somewhat counterintuitively, adolescent subjects displayed less sign tracking and more goal tracking than adults. Indeed, the distribution of PCA index for adolescents was significantly shifted to the negative side (*p* = 0.002, Wilcoxon signed rank test), while the adult distribution was significantly shifted positive (*p* = 0.003), indicating an overall preference for sign tracking among the adult population and goal tracking among adolescents.

After the completion of Pavlovian conditioned approach training, we trained adolescent rats on the rGT with win cues, a task originally adapted for our lab from Barrus and Winstanley (2016). In this task, rats chose among three nosepokes that were associated with different probabilities of reward (1–4 sugar pellets) or timeout (5–40 s). Reward delivery was accompanied by audiovisual cues that increased in salience along with reward size. For all rats, nosepoke 1 (NP1) was the “safe” choice (low risk, low reward), NP2 was moderate (medium risk, medium reward), and NP3 was the risky choice (high risk, high reward). The optimal choice was NP2, in that consistently choosing NP2 would result in the greatest quantity of rewards obtained over the fixed time (40 min) session.

Adolescents’ behavior, like that of adults, was essentially stable after 7 days of training on the full task: a repeated measures ANOVA found no interaction of session and choice percentages over days 5–7 of training (*F*_(4, 378)_ = 0.47, *p* = 0.76). However, we found that fully trained adolescents and adults exhibited markedly different patterns of choice on the rGT (Figures 2E,F; age x choice, *F*_(2, 252)_ = 12.60, *p* < 0.001). Averaged over the last three days of training, adolescents made significantly more risky choices (*p* < 0.001) and significantly fewer optimal choices (*p* = 0.004), as well as slightly fewer safe choices (*p* = 0.04), compared with adults. This is consistent with many studies demonstrating that adolescents have a tendency toward high-risk, high-reward choices, compared with adults, across species (Cauffman et al., 2010; Doremus-Fitzwater et al., 2010; Westbrook et al., 2018).

### Relationship of sign tracking to risky choice

**Figure 3:**
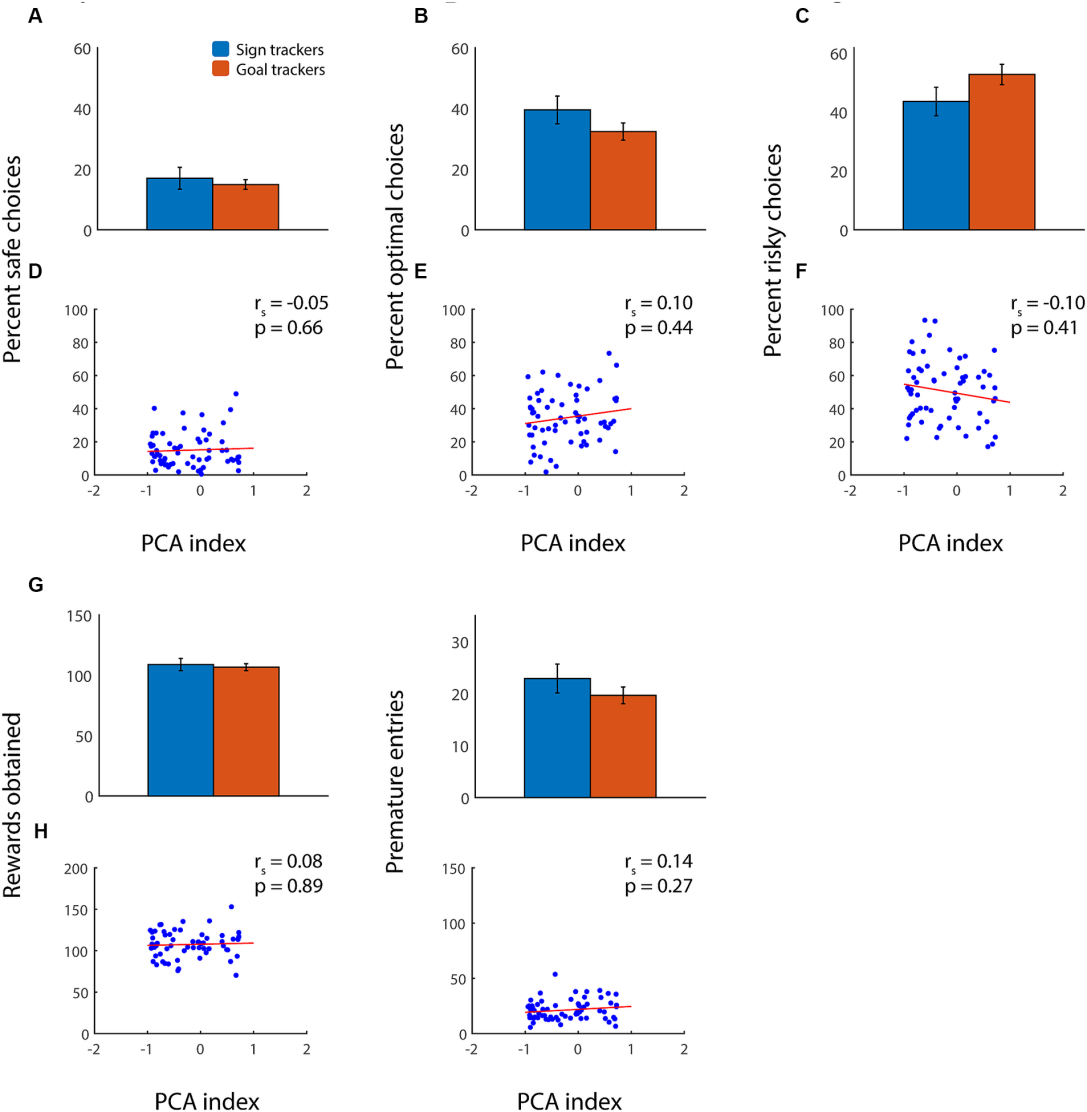
Adolescents’ sign tracking or goal tracking behavior is not correlated with performance on the rGT with win cues. **(A–C)** Average percent choice of the safe option **(A)**, optimal option **(B)**, or risky option **(C)** among adolescent sign trackers (blue) and goal trackers (orange) performing the rGT. **(D–F)** Average percent choice of safe **(D)**, optimal **(E)**, and risky **(F)** options for each subject plotted as a function of PCA index. **(G,H)** Average total rewards obtained **(G)** and premature nosepokes **(H)** among adolescent sign trackers (blue) or goal trackers (orange). Regression lines in red.

We previously showed that, in adult rats, a propensity toward sign tracking predicted suboptimal performance on the rGT with win cues (Swintosky et al., 2021). A higher PCA index was associated with increased risky choices, decreased optimal choices, somewhat more premature nosepokes, and, overall, fewer rewards obtained. We hypothesized that the relationship between sign tracking and/or goal tracking and risky decision-making would be different in adolescents, given their distinct behavioral profile in these tasks. Indeed, in contrast to adults, we found little or no relationship between adolescents’ tendency toward sign-tracking or goal-tracking behavior and their performance on the rGT (Figure 3). In adolescent animals, there was no correlation between PCA index and safe, optimal, or risky choices (Figures 3A–F), nor did PCA index predict the number of rewards obtained (Figures 3G,H). Unexpectedly, there was also no relationship between PCA index and premature nosepokes (Figures 3I,J). These findings were similar whether we examined point-by-point correlations or performed a binary comparison of sign trackers with goal trackers (all comparisons, *p* > 0.15, Wilcoxon rank sum test).

Because PCA index is a composite measure, it could be masking subtler relationships between individual behaviors. Therefore, we also analyzed the relationship between number and latency of lever presses or magazine entries during Pavlovian conditioning with choices, rewards, and premature entries during the rGT: none of these showed any significant correlation (*p* > 0.15 for Spearman’s rho in all cases; data not shown). Similarly, there was no relationship between any of these parameters and latency to choose any of the options in the rGT. We concluded that, unlike in adult animals, adolescents’ sign-tracking behavior was not predictive of suboptimal performance during the rGT. Indeed, adolescents with a strong goal-tracking behavioral profile made as many or more high-risk, high-reward choices as adolescents with a strong tendency toward sign-tracking.

### Behavior of adults trained during adolescence

Some previous studies have suggested that the age at which subjects are first exposed to gambling – adolescent or adult – can have profound effects on adult behavior, including risky decision-making and impulsive action (Rahman et al., 2012; Cho et al., 2018). Therefore, we retested the behavior of a subset of adolescents trained on the rGT (*n* = 32) after they reached adulthood. The age of this group at retest (13–14 weeks) was similar to that of a comparison group of adults trained during adulthood (Swintosky et al., 2021). The baseline sign tracking and/or goal tracking behavior of the retested group was not different from the group that was not retested (*p* = 0.6, chi-square test).

**Figure 4:**
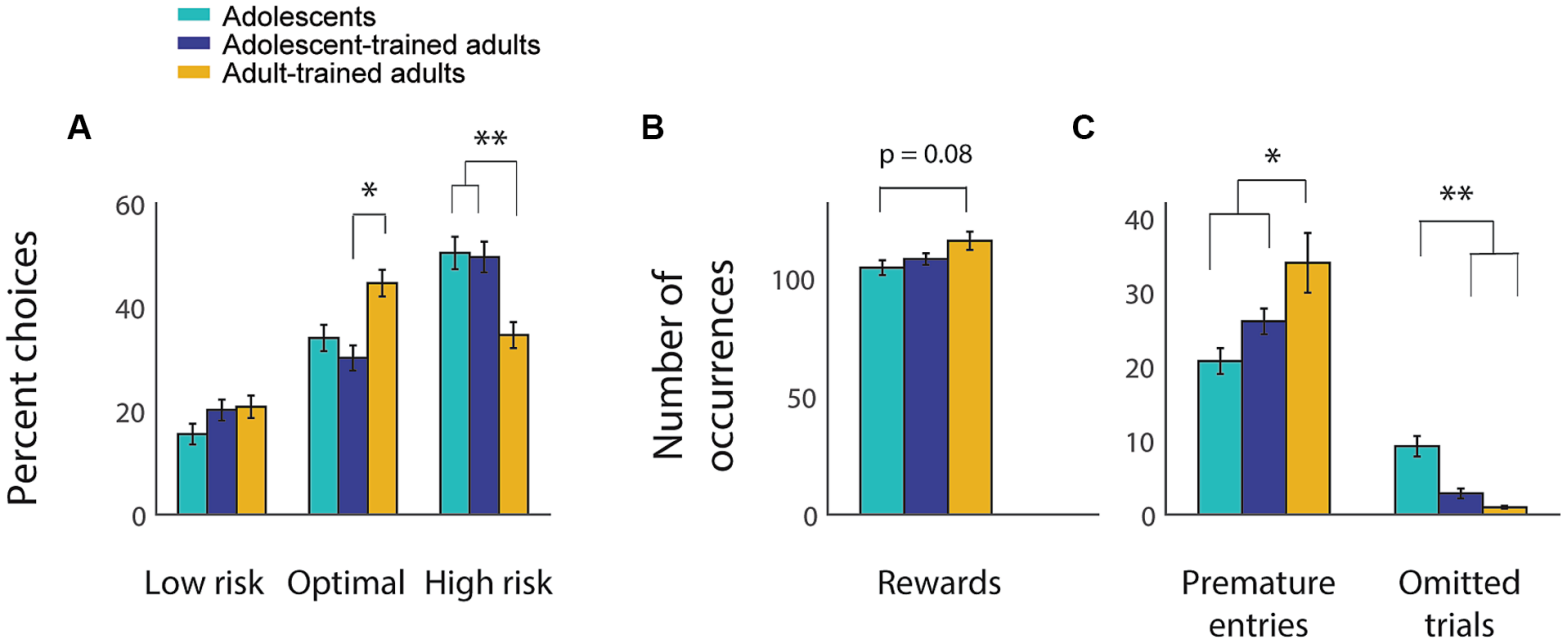
Adults who were trained on the rGT as adolescents retain an adolescent-like choice profile. **(A–C)** Average choice distribution **(A)**, number of rewards obtained **(B)**, and numbers of premature nosepokes and omitted trials **(C)** for adolescent-trained animals as adolescents (light blue) and adults (dark blue) and for a separate population of adults trained during adulthood (yellow). Asterisk, *p* < 0.05; double asterisk, *p* < 0.001. Error bars indicate SEM.

To our surprise, we found that adults exposed to the rGT as adolescents retained behavior that was almost indistinguishable from that of adolescents, and that was quite different from that of adults trained during adulthood (Figure 4). A two-way repeated measures ANOVA found no significant interaction between age (adolescent vs. adult) and choice percentage for rats trained as adolescents (*F*_(2, 124)_ = 0.95, *p* = 0.39). However, when adult-trained adults were included, a two-way ANOVA revealed a significant interaction between group and choice (Figure 4A; *F*_(4, 383)_ = 9.99, *p* < 0.001), with adolescent-trained adults making significantly fewer optimal choices (*p* = 0.02) and significantly more risky choices (*p* = 0.004) than adult-trained adults. Despite making more optimal choices, adult-trained animals did not obtain significantly more rewards overall compared to the adolescent-trained groups (Figure 4B; *F*_(2, 127)_ = 2.56, *p* = 0.08). This is likely due to differences in the number of premature nosepokes (Figure 4C; *F*_(2, 127)_ = 3.55, *p* = 0.03): because premature nosepoke entries resulted in a brief timeout, each premature entry reduced the time available for obtaining rewards. Adult-trained adults displayed significantly more premature nosepokes than adolescents (*p* = 0.03), whereas adolescent-trained adults did not (*p* = 0.64).

One way that adolescent-trained adults were more similar to other adults than to adolescents was with regard to omitted trials (Figure 4C). Both adolescent- and adult-trained adults had very few omitted trials, while adolescents omitted significantly more (*F*_(2, 127)_ = 36.98, p < 0.001; adolescents vs. either adult group, p < 0.001). Overall, adolescent-trained adults showed a choice profile more similar to adolescents than to adults; a level of premature action that was between that of adolescents and adults; and a level of omitted trials that was similar to other adults.

## Discussion

Sign tracking behavior is thought to represent an individual’s tendency to transfer incentive salience from a reward to a reward-predictive cue (Flagel et al., 2009). In adult male rats, a propensity toward sign tracking is related to suboptimal behavior on a rodent gambling task with win cues, including both maladaptive risky choices and impulsive, premature actions (Swintosky et al., 2021). Both of these behaviors contribute to adult sign trackers earning fewer rewards than goal trackers over the course of a gambling session. Here, we show that the same relationship does not hold true for adolescent rats: young rats with a propensity toward sign tracking, compared with young goal trackers, do not exhibit higher rates of risky choice or premature nosepoke entry, and do not obtain fewer rewards overall. This result points to key differences in cue reactivity, risky decision-making, impulsivity – or all three – based on developmental stage, suggesting changes over the course of development in the way the neural circuits underlying these behaviors become engaged and interact.

The current study is the first, to our knowledge, to show that adolescent rats prefer the high-risk, high-reward option on a version of the rGT; compared with adults, they make substantially more high-risk choices, primarily at the expense of optimal choices. This finding is consistent with many studies showing that, across species, adolescents exhibit heightened risk-taking behavior relative to children and adults (Steinberg, 2008; Doremus-Fitzwater et al., 2010; Zoratto et al., 2013; Defoe et al., 2015; Westbrook et al., 2018). More specifically, the current study replicates in a rodent model the finding that human young adolescents perform suboptimally on the Iowa Gambling Task (Marquez-Ramos et al., 2023), making more maladaptive choices of the high-risk, high-reward deck than adults. Therefore, this finding extends the translational utility of the rGT, which has already been well validated in adult rats (Zeeb et al., 2009; Clark et al., 2013; Barrus and Winstanley, 2016), to an adolescent population. The rGT with win cues may also be considered as a semi-realistic model of a typical gambling environment, complete with attention-grabbing audiovisual stimuli; in this context, the current study lays the groundwork for future translational studies using the rGT to examine the neural basis of adolescent risk-taking and gambling, including pathological gambling, which is a growing public health problem (Calado et al., 2017; Richard and King, 2023).

The current study also replicates the finding by multiple groups that adolescent rats exhibit substantially less sign tracking and more goal tracking than adults (Anderson and Spear, 2011; Doremus-Fitzwater and Spear, 2011; Anderson et al., 2013; Rode et al., 2019). This implies that adolescents are less likely than adults to transfer incentive salience from a reward to a cue, at least in the context of Pavlovian conditioning. Indeed, we have demonstrated that goal tracking in adolescent rats is genuinely a goal-oriented behavior – not simply an alternate manifestation of sign tracking – by showing that adolescent goal tracking, unlike sign tracking, is very sensitive to reward devaluation (Rode et al., 2019), just as it is in adults (Morrison et al., 2015; Patitucci et al., 2016). Resistance to reward devaluation is commonly used as an operational definition of habitual behavior (Balleine and O'Doherty, 2010), although sign tracking is not considered a habit, *per se*, because it arises from a Pavlovian rather than an instrumental response (Dayan and Berridge, 2014). However, it is possible that reduced sign tracking in adolescents is related to their weaker tendency toward habit formation (Serlin and Torregrossa, 2015; Towner et al., 2020): both are consistent with adolescents’ enhanced cognitive and behavioral flexibility (Simon et al., 2013; Westbrook et al., 2018) and exploratory drive (Douglas et al., 2003).

Adolescence is a time when animals must learn the rules, including reward contingencies, of their environment, so it may be advantageous to avoid habit formation and forms of “automatic” cue-driven behavior that could interfere with exploration and learning. Similarly, although risky decision-making often results in suboptimal outcomes (including in the rGT), a heightened tolerance for risk in adolescence might be a necessary tradeoff for fostering independence and exploration. With regard to the underlying mechanisms of adolescent risk-taking, the “dual systems” model (Steinberg et al., 2008; Shulman et al., 2016), based on human subject data, posits that a fast-maturing incentive processing system promotes reward-, sensation-, and novelty-seeking among adolescents, while an immature cognitive control system cannot fully restrain impulsive behavior. Likewise, many studies have demonstrated that adolescent rodents show heightened reward sensitivity paired with weaker sensitivity to aversive outcomes (Friemel et al., 2010; Zoratto et al., 2013; Doremus-Fitzwater and Spear, 2016). These characteristics might be sufficient to account for adolescents’ high-risk, high-reward choice preference in the rGT, regardless of the influence of reward-associated cues.

In adults, on the other hand, evidence suggests that risky choices are at least partially driven by salient reward-related cues. The presence of “win cues” in a gambling task increases the proportion of risky choices in both rats (Barrus et al., 2016; Barrus and Winstanley, 2016) and humans (Cherkasova et al., 2018). Moreover, the association between sign tracking and risky decision-making (Swintosky et al., 2021) implies that the cue reactivity of individual subjects plays a role in determining their choices in the rGT. Further research is needed to determine whether this is the case specifically when rewards are paired with salient audiovisual cues, or if sign tracking is predictive of high-risk, high-reward choices more generally. Interestingly, sign tracking was also associated with premature nosepoke entries in adults (Swintosky et al., 2021) but not adolescents, and adults performed more premature entries than adolescents overall. This finding is at odds with the idea that adolescents are more impulsive than adults in general, although this is certainly the case on a number of tasks (Burton and Fletcher, 2012; Jadhav et al., 2022). There may be situations, including the current task, when reward-associated cues elicit impulsive actions from adults more readily than from adolescents. The influence of cues (or relative lack of such) might explain why adolescents measure higher than adults on one dimension of impulsivity (maladaptive risky choices) but lower on another (premature actions). Again, additional studies are needed to clarify whether this finding is specific to the rGT with win cues or generalizes across other risky decision-making tasks.

Moreover, it is important to note that the current study was limited to male rats, and that their gambling-like behavior was assessed only in late adolescence (by necessity, given the duration of training required). In human adolescents, both of these factors – male sex and older age – are positively associated with problem gambling (Dowling et al., 2017; Richard and King, 2023), and human young adult males are more likely than females to show signs of GD (Wong et al., 2013). However, in adult rats, studies have found that sex differences in performance on the rGT are absent or subtle at baseline, depending on the specific analysis (Georgiou et al., 2018; Hynes et al., 2020), although male and female rats vary in their response to dopaminergic manipulations during this task (Hynes et al., 2021, 2023). Further research is needed to examine possible sex differences among adolescents in risky decision-making during the rGT.

Although we expected that early exposure to gambling might have an effect on subsequent adult behavior, we were surprised to find that adults trained on the rGT as adolescents retained a choice profile that was virtually indistinguishable from adolescents. Few previous animal studies have examined early vs. late exposure to gambling or other risky decision-making, although one report (Cho et al., 2018) found that early training on a version of the rGT was associated with increased impulsive action, but not impulsive choice, in later adulthood. This finding contrasts somewhat with the current study; for example, we did not see increased premature entries in adolescent-trained adults compared with adult-trained adults. However, there are several differences in methodology that might account for this disparity: in particular, in the study of Cho et al., rats were not exposed to the full rGT until young adulthood (about 77 days of age). Meanwhile, human epidemiological studies have shown that age of exposure to gambling is related to the later probability and severity of pathological gambling (Rahman et al., 2012; Dowling et al., 2017). Moreover, a substantial body of literature shows that adolescent drug use in rodents – especially exposure to alcohol or THC – is related to later maladaptive risky decision-making (Nasrallah et al., 2009; Clark et al., 2012; McMurray et al., 2016; Ferland et al., 2023). Because problem gambling and substance disorders share overlapping neural substrates (Hynes et al., 2021), it is possible that early exposure to gambling and early exposure to drugs of abuse might result in similar changes in adult risk-taking behavior.

Taking this evidence into account, it is reasonable to speculate that exposure to a gambling environment in adolescence predisposes subjects to make maladaptive risky choices as adults. However, an important caveat is that, in the current study, adolescents were exposed not only to the rGT, but also to Pavlovian conditioning designed to elicit sign tracking and/or goal tracking. It is possible that exposure to Pavlovian conditioning in early adolescence, or to a combination of the two tasks, might have influenced risky decision-making in adulthood. Alternatively, or in addition, it is possible that adolescent-trained adult rats might be influenced by memory or familiarity with the rGT, possibly in the form of persistent stimulus-action associations. In other words, because individuals were motivated to pursue high-risk, high-reward choices in a specific context as adolescents, they might perseverate on such choices as adults when placed in the same context. Future studies must determine if early exposure to a gambling task, such as the rGT, affects risky decision-making on different tasks, or even the same task in a different context or environment, during adulthood.

Overall, the current findings point to ways in which adolescent gambling behavior might rely on different underlying neurobehavioral processes compared with adult gambling. In particular, the evidence suggests that adolescents may be less influenced by some kinds of reward-associated cues than adults, as reflected by their weaker sign tracking behavior (Anderson et al., 2013; Rode et al., 2019), while at the same time being more acutely sensitive to primary rewards, such as palatable foods (Friemel et al., 2010), and less sensitive to aversive outcomes (Doremus-Fitzwater and Spear, 2016), making them more risk-tolerant (Zoratto et al., 2013). We also highlight the possibility of early exposure to gambling leading to changes in subsequent adult risk-taking behavior – perhaps analogous to early exposure to drugs of abuse. All of these findings are relevant for understanding, and ultimately ameliorating, adolescent gambling, which is a growing public health problem (Richard and King, 2023). More generally, we establish that the rGT with win cues can be a useful translational model of gambling behavior for adolescent rats in addition to adults (Barrus and Winstanley, 2016; Winstanley and Clark, 2016). Additional research can build on these findings to investigate the neural circuitry and neurochemistry underlying adolescent vs. adult risk-taking behavior in the rGT.

## Data availability statement

The raw data supporting the conclusions of this article will be made available by the authors, without undue reservation.

## Ethics statement

The animal study was approved by the University of Pittsburgh IACUC. The study was conducted in accordance with the local legislation and institutional requirements.

## Author contributions

SZ: Data curation, Investigation, Writing – review & editing. EM: Data curation, Investigation, Writing – review & editing. AR: Data curation, Investigation, Writing – review & editing. SM: Conceptualization, Formal analysis, Funding acquisition, Supervision, Visualization, Writing – original draft.
